# Society of Skeletal Radiology– white paper. Guidelines for the diagnostic management of incidental solitary bone lesions on CT and MRI in adults: bone reporting and data system (Bone-RADS)

**DOI:** 10.1007/s00256-022-04022-8

**Published:** 2022-03-28

**Authors:** Connie Y. Chang, Hillary W. Garner, Shivani Ahlawat, Behrang Amini, Matthew D. Bucknor, Jonathan A. Flug, Iman Khodarahmi, Michael E. Mulligan, Jeffrey J. Peterson, Geoffrey M. Riley, Mohammad Samim, Santiago A. Lozano-Calderon, Jim S. Wu

**Affiliations:** 1 Massachusetts General Hospital, Boston, USA; 2 Mayo Clinic Florida, Jacksonville, USA; 3 Johns Hopkins University, Baltimore, USA; 4 MD Anderson Cancer Center, Houston, USA; 5 University of California San Francisco, San Francisco, USA; 6 Mayo Clinic Arizona, Phoenix, USA; 7 New York University, New York, USA; 8 University of Maryland, College Park, USA; 9 Stanford Medical Center, Palo Alto, USA; 10 Beth Israel Deaconess Medical Center, Boston, USA

**Keywords:** Bone tumors, MRI, CT, Management

## Abstract

The purpose of this article is to present algorithms for the diagnostic management of solitary bone lesions incidentally encountered on computed tomography (CT) and magnetic resonance (MRI) in adults. Based on review of the current literature and expert opinion, the Practice Guidelines and Technical Standards Committee of the Society of Skeletal Radiology (SSR) proposes a bone reporting and data system (Bone-RADS) for incidentally encountered solitary bone lesions on CT and MRI with four possible diagnostic management recommendations (Bone-RADS1, leave alone; Bone-RADS2, perform different imaging modality; Bone-RADS3, perform follow-up imaging; Bone-RADS4, biopsy and/or oncologic referral). Two algorithms for CT based on lesion density (lucent or sclerotic/mixed) and two for MRI allow the user to arrive at a specific Bone-RADS management recommendation. Representative cases are provided to illustrate the usability of the algorithms.

## Introduction

Incidental solitary bone lesions are frequently encountered on computed tomography (CT) and magnetic resonance imaging (MRI) in routine clinical practice. Despite the prevalence of these unexpected lesions, clear and consistent guidelines for their management have not been defined in the literature [1]. Guidance on differentiating lesions, which can be safely ignored, require additional imaging, follow-up, or tissue sampling benefits radiologists, and in particular non-musculoskeletal (MSK) radiologists. The purpose of this white paper is to present algorithms for the diagnostic management of solitary bone lesions incidentally encountered on CT and MRI in adults. Pediatric patients have a unique set of lesions and a unique appearance of the bone and bone marrow, and therefore are not specifically addressed in this paper.

The Practice Guidelines and Technical Standards Committee of the Society of Skeletal Radiology (SSR) identified management of incidental bone lesions as an important topic for study. Twelve SSR members and one orthopedic oncologist with expertise in bone tumor imaging formed an ad hoc white paper committee to develop evidence-based diagnostic management algorithms for incidental solitary bone lesions encountered on CT and MRI. The committee tested the algorithms using sample case scenarios, and the algorithms were revised in consensus after additional debate. The algorithms were presented to the SSR membership at the SSR 2021 annual meeting for written comments. An additional open forum SSR member discussion session with the panel members was conducted. SSR member comments and suggestions were taken into consideration for incorporation into the final manuscript.

**Fig. 1:**
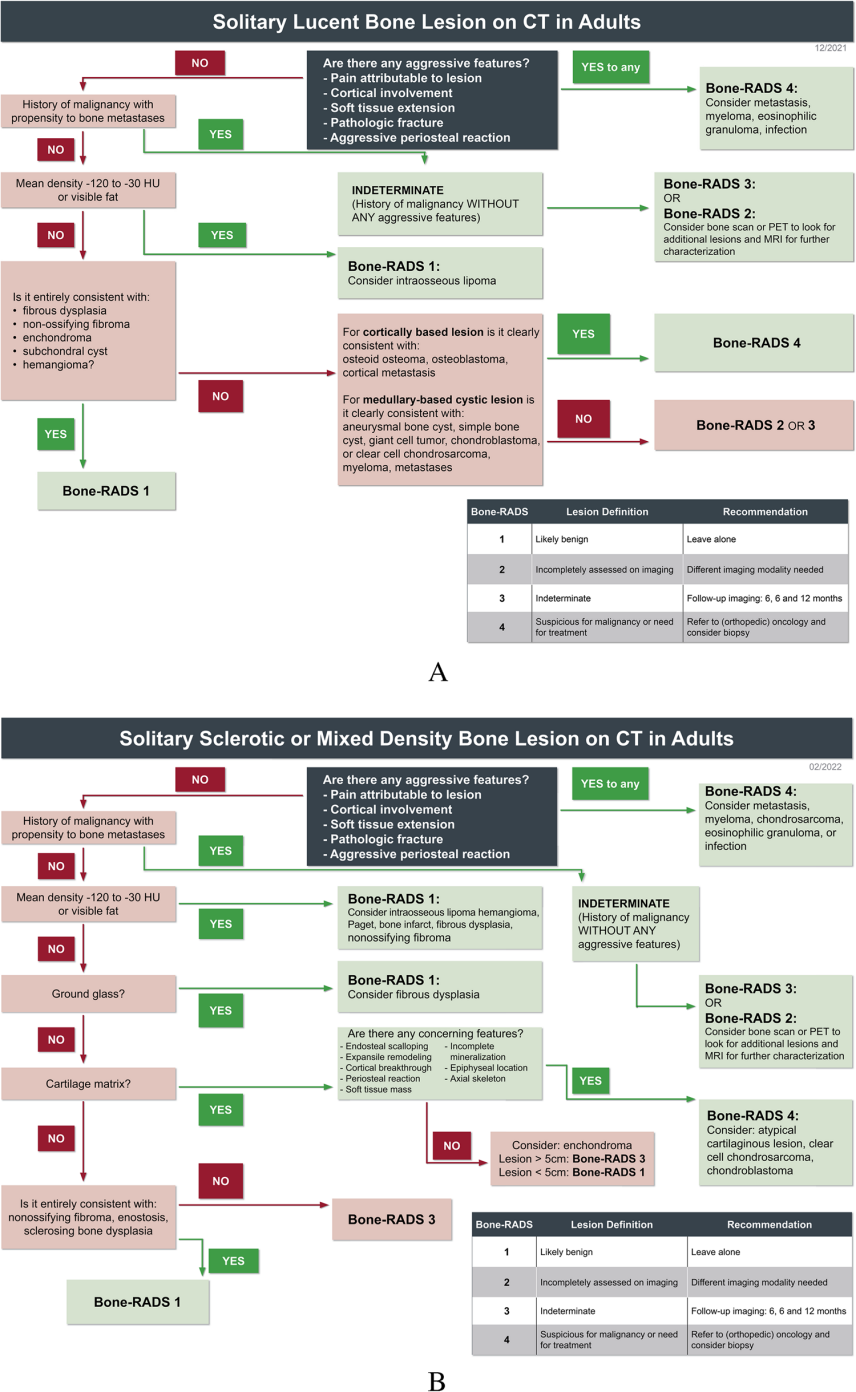

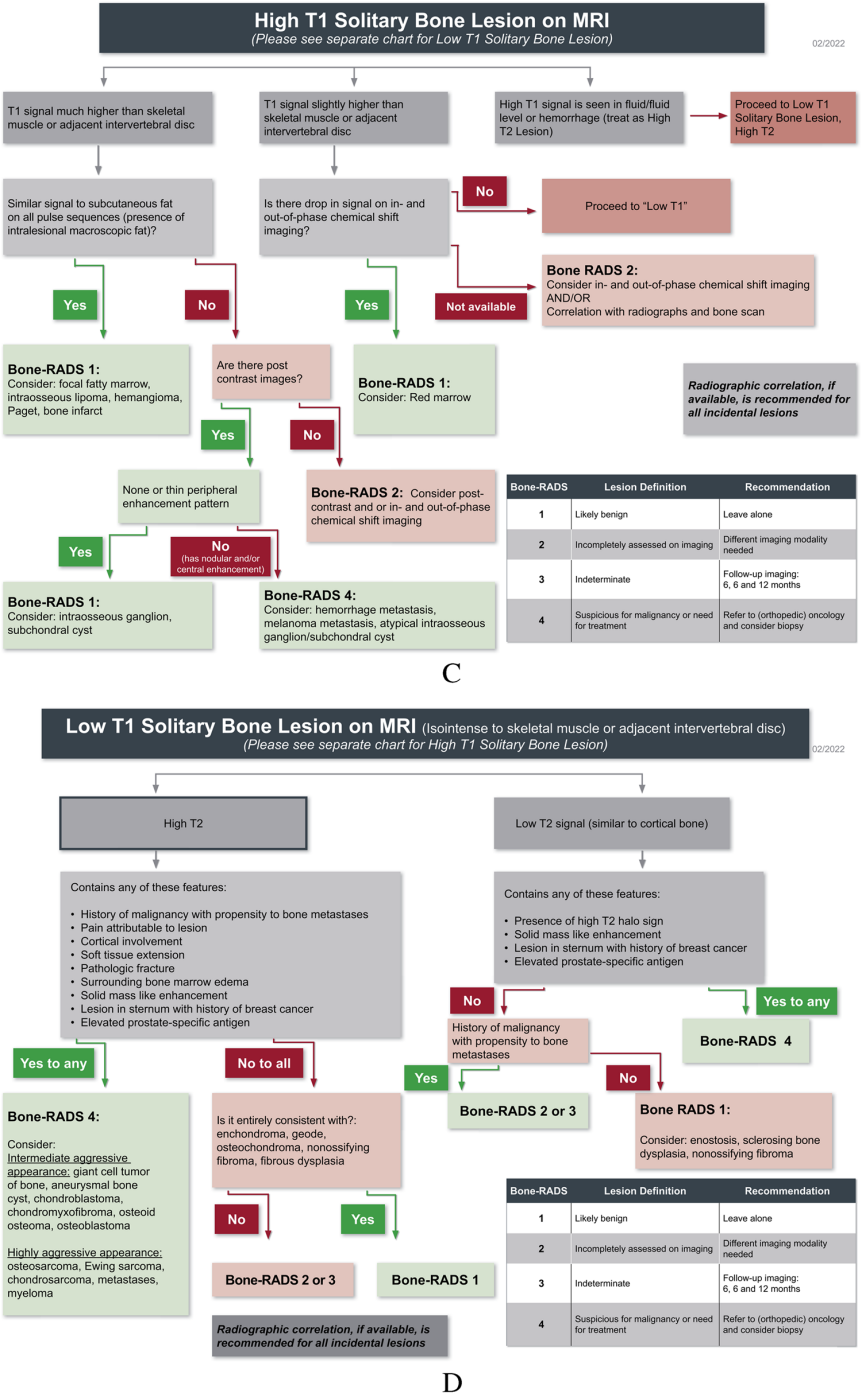
Flowcharts for evaluating: **A** solitary lucent bone lesions on CT, **B** solitary sclerotic and mixed density bone lesions on CT, **C** High T1 solitary bone lesion on MRI, and **D** low T1 solitary bone lesion on MRI

This consensus statement summarizes the current understanding of incidentally encountered bone lesions on CT and MRI. It is important to note that the algorithms (Fig. 1) presented focus on the diagnostic management of incidental bone lesions instead of arriving at a specific diagnosis. The guidelines and recommendations outlined in this paper are intended for use by all radiologists; however, given the incidental nature of these lesions, may be more commonly used outside of a musculoskeletal practice.

## Scope and definitions

### Scope: Development of Bone-RADS

We have chosen to focus on solitary incidental bone lesions discovered on CT and MRI as we feel these lesions have the highest degree of inconsistency in diagnostic management. Lesions encountered on radiography can be assessed for aggressive or non-aggressive features including bone destruction, lesion margin, tumor matrix, and periosteal reaction, and their management is well documented [2–6]. Many of these radiographic features can also be assessed on CT and have been incorporated into the algorithms. However, radiographic and CT features are not easily translatable to lesions encountered on MRI. Therefore, a separate algorithm based on MRI signal characteristics is developed here. We limited our scope to solitary lesions because patients with multiple lesions often have metastatic disease or systemic conditions that typically require biopsy and/or oncologic referral. Of note, standardization of imaging technique and more advanced clinical management decisions, such as surgery, radiation, chemotherapy, and ablation therapy, are beyond the scope of our intent. Finally, these algorithms are intended to provide a framework for evaluation, and not to override expert opinion or clinical context.

### When is a bone lesion “incidental”?

Radiologists routinely interpret imaging studies that include bone lesions unrelated to the presenting clinical concern. The Oxford English Dictionary provides one definition of “incidental” as “occurring by chance in connection with something else.” The term “incidental” has become common lexicon in radiology, and we are defining it in this article as “a lesion detected on an imaging study performed for an unrelated reason.” An incidental lesion can be definitively irrelevant, of uncertain clinical significance requiring further workup, or concerning requiring treatment, such as a malignancy or infection.

### CT classification of bone lesions: lucent, sclerotic, or mixed density

#### Definition of “lucent”

**Fig. 2:**
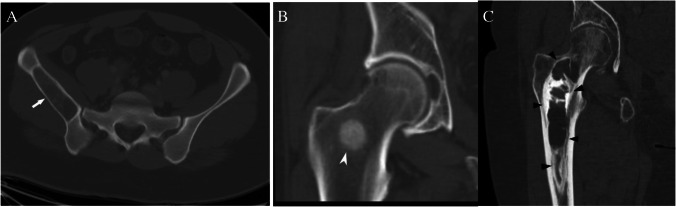
CT classification of bone lesions by density. CT images show **A** lucent (arrow), **B** sclerotic (pointed arrow), and **C** mixed density (arrowheads) lesions in 3 patients with giant cell tumor, prostate cancer metastasis, and fibrous dysplasia, respectively

Lucent lesions are commonly encountered; however, a well-defined quantitative description has not been established. For the purposes of this paper, a “lucent” lesion is defined as a lesion that replaces and has lower attenuation than normal trabecular bone, resulting in a hypodense CT appearance. A lesion is defined as lucent if greater than 90% of the volume of the lesion qualifies as lucent (Fig. 2A) and should be evaluated using the “lucent lesion” flowchart. On CT imaging, this includes lesions that contain solid non-sclerotic areas of soft tissue attenuation, such as giant cell tumor, or cystic lesions containing fluid or blood products, such as an aneurysmal bone cyst [7, 8]. There is no consistent recommendation for a Hounsfield unit (HU) definition of a lucent lesion in the literature; however, fat can have an attenuation between − 120 and − 30 HU [9]. Non-fatty lucent lesions have HU values between 0 and 200. Osteoporotic trabecular bone typically is around 120 HU and normal trabecular bone up to 200 HU [10]. Gout tophus can be around 160 HU [11]. “Ground glass” attenuation of fibrous dysplasia can have a wide range but is typically > 100 HU but well less than 885 HU.

#### Definition of “sclerotic” or “mixed density”

For bone lesions, the terms “sclerosis,” “sclerotic,” “osteosclerotic,” or “osteoblastic” are broadly used during interpretation of radiographs or CT to describe a bone lesion with higher density or attenuation than the surrounding or adjacent trabecular bone. There is currently no specific attenuation range that clearly defines “sclerosis” or sclerotic,” so these terms are otherwise nebulous and are subjectively applied by the interpreter. To our knowledge, the only defined classification that has been used in the literature was part of an informal methodology prescribed by Leffler and Chew when they investigated the accuracy of percutaneous CT-guided biopsy for diagnosis of sclerotic bone lesions [12]. In their methods, the authors describe a density grading system with grades 1–4 relative to cortical bone, with grade 1 being “barely perceptible” and grade 4 being “denser than cortex.” This informal classification has not been widely adopted since it was published in 1999. Other definitions of “sclerosis” reported more recently in the literature define a “dense” sclerotic lesion as one where ≥ 50% of its volume is denser than the surrounding normal trabecular bone and “mixed” if the lesion does not reach this threshold [7, 13–15]. The most common lesions that qualify as “sclerotic” are enostoses and osteoblastic metastases (Fig. 2B) [16].

There is also no widely accepted definition for a “mixed” density lesion, but the term is commonly used to describe a lesion that contains a combination of osteoblastic (sclerotic) and osteolytic (lucent) areas relative to adjacent trabecular bone on CT. The ratio of sclerosis to lucency defining a “mixed” lesion remains subjective. For the purposes of this white paper, a lesion is considered a “mixed” lesion when it has equivalent or near equivalent amount of sclerosis and lucency (1:1 ratio). There are several bone lesions that have been traditionally considered “mixed” in the experience of the authors, including benign fibro-osseous lesions, chondroid lesions, osteonecrosis, and degenerative periarticular lesions such as subchondral cysts (Fig. 2C).

For the purposes of this white paper, we have grouped sclerotic and mixed density lesions together in a single flowchart. Any lesion that does not fit the above definition of a “lucent” lesion should be evaluated as a sclerotic/mixed density lesion.

### MRI classification of bone lesions

#### Definition of T1 hyperintense lesions

**Fig. 3:**
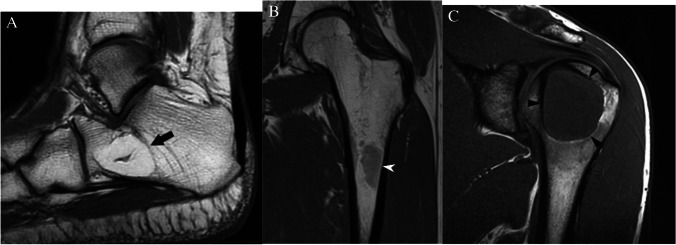
MRI lesions based on T1 signal intensity. T1-weighted MRI images show **A** a calcaneal intraosseous lipoma (arrow) that has signal intensity much higher than skeletal muscle with the same signal intensity as fat, **B** a focus of red marrow (arrowhead) in the proximal femur that is slightly hyperintense to skeletal muscle, and **C** a chondroblastoma (arrowheads) in the humeral that has signal isointense to skeletal muscle

Accurate assessment of the T1 signal intensity is of primary importance when determining the nature of an incidental bone lesion. If no T1-weighted sequence is available, we recommend that the patient return for additional T1-weighted images. For our purposes, T1 hyperintense bone lesions are defined as lesions that demonstrate T1 signal that is visually higher signal intensity than the T1 signal of adjacent skeletal muscle or intervertebral disc [17]. Some of these lesions can have T1 signal “much higher” than skeletal muscle or intervertebral disc due to macroscopic fat such as an intraosseous hemangioma (Fig. 3A), whereas other lesions have signal only “slightly higher” such as hematopoietic (red) marrow (Fig. 3B). Of course, some infiltrative lesions such as lymphoma or leukemia can still maintain some intralesional fat, and some vascular lesions may be T1 hyperintense to surrounding muscle, but these lesions are not typically solitary or incidental.

#### Definition of T1 isointense/hypointense lesions

T1 isointense/hypointense bone lesions are defined as lesions that demonstrate T1 signal that is the same or lower than the T1 signal of adjacent skeletal muscle or intervertebral disc (Fig. 3C). Most bone tumors and metastasis fall into this category and necessitate assessment of their T2 characteristics to further discriminate between clearly benign lesions and those that that require further workup. These definitions are based on subjective visual assessment alone and do not incorporate the use of an intralesional region of interest for quantitative value determination.

#### Definition of T2 hypointense and hyperintense lesions

**Fig. 4:**
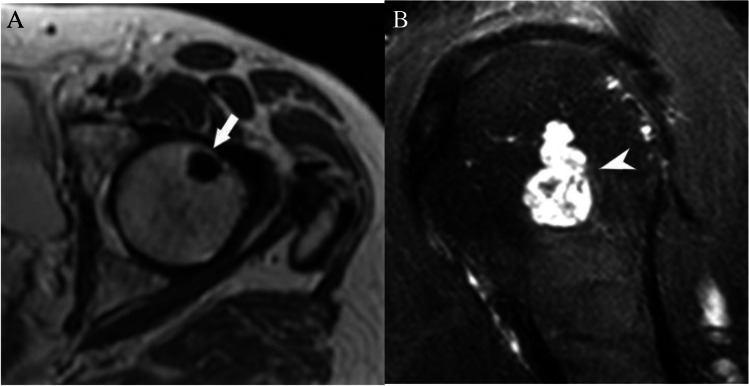
MRI lesions based on T2 signal intensity. **A** Axial T2-weighted MRI image of the hip shows a uniformly hypointense lesion (arrow) in the femoral head consistent with an enostosis. **B** Sagittal T2-weighted fat-suppressed MRI images show a lobulated hyperintense lesion in the humeral head consistent with a low-grade cartilage lesion/enchondroma

Many benign and malignant bone lesions have a higher free water content than the surrounding normal marrow fat and therefore are higher signal intensity than marrow fat on T2-weighted imaging. Most MR protocols in use today employ methods to accentuate this brightness differential by suppressing the T2 signal of the fat, by using either fat saturated or short tau inversion recovery (STIR) sequences. For the purposes of this white paper, T2 hypointense is used to describe lesions with little to no free water that are similar in T2 signal to air or cortical bone, skeletal muscle or fat which has been adequately fat suppresses, including enostoses, and osteoblastic metastases (Fig. 4A). These lesions are lower in signal than fat on fat-suppressed images. Conversely, T2 hyperintense is used to describe lesions that have very high signal similar to fluid (joint effusion, bladder, CSF) (Fig. 4B), including enchondromas and simple bone cysts. Intermediate T2 signal is less hyperintense than fluid but clearly more hyperintense than skeletal muscle or suppressed fat signal.. These definitions can be used in both T2 and T2 fat-suppressed images.

## Management options: Bone-RADS

The committee proposes a bone reporting and data system (Bone-RADS) with four possible diagnostic management recommendations (Table 1): Bone-RADS1, leave alone; Bone-RADS2, perform different imaging modality; Bone-RADS3, perform follow-up imaging; and Bone-RADS4, biopsy and/or oncologic referral. If a lesion is clearly benign, such as in the case of an enostoses or non-ossifying fibroma, then it can and should be left alone (Bone-RADS1). If the lesion has clearly concerning features (large soft tissue component, intralesional enhancement) or is at risk for pathologic fracture, then the lesion needs to undergo biopsy and/or oncologic evaluation (Bone-RADS4). For indeterminate lesions, two options exist for the radiologist. The first option is to obtain different imaging to provide additional diagnostic information (Bone-RADS2), and the second option is to repeat the same imaging after a certain time period (Bone-RADS3) to assess for increase in size or development of worrisome features, such as cortical involvement. The recommendation is for Bone-RADS3 lesions 3 to undergo follow-up at 6,6,12 months intervals for a total of 2 years. For any bone lesion, one of these four management options should be selected. A major goal of the panel when creating these algorithms was to be certain that lesions designated Bone-RADS1 are truly benign processes that require no additional workup, such as a non-ossifying fibroma, enostoses, or focus of red marrow. Thus, the algorithms are weighted towards identifying Bone-RADS1 lesions.

**Table 1:** BONE-RADS categories for incidental bone lesions

Category	Lesion definition	Recommendation
1	Likely benign	Leave alone
2	Incompletely assessed on imaging	Different imaging modality needed
3	Indeterminate	Follow-up imaging: 6, 6, and 12 mos
4	Suspicious for malignancy or need for treatment	Biopsy and/or referral to orthopedic oncology

**Fig. 5:**
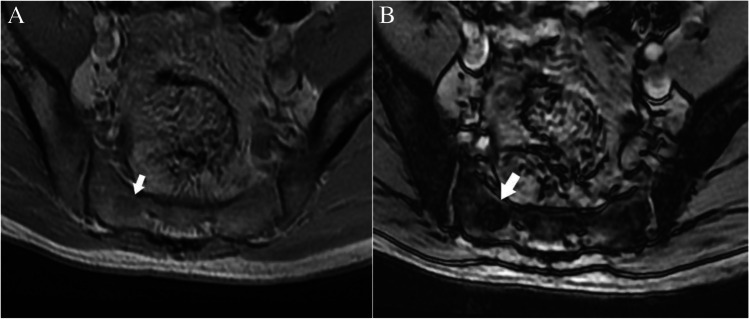
Chemical shift imaging. 56-year-old man with incidental lesion in the right sacral ala. The lesion (arrows) is isointense to skeletal muscle on the **A** in-phase T1-weighted gradient echo MR image and has uniform loss (drop) in signal on the **B** oppose phase T1-weighted gradient echo MR image indicating the presence of internal fat. The lesion is compatible with a focus of red marrow

Three management algorithms are provided, two for CT lesions (“lucent” and “sclerotic/mixed”) and one for MRI. Both CT and MRI have useful features that can be used to aid the radiologist in arriving at the most appropriate recommendation. The CT algorithms incorporate lesion density, in Hounsfield units (HU), since very dense lesions (greater than 885 HU) and lesions containing fat (less than − 30 HU) are often benign [18–21]. Please note that there is some variation in the literature about the density range of fat in the literature. The most commonly accepted range is − 120 to − 30 HU, although one study has − 10 HU at its upper limit. Also, there is likely variability between patients, among different locations of the body, even within the same patient, and also due to imaging technique on different scanners, and therefore comparison to surrounding normal fat is often helpful [22–24]. The authors recognize that assessing the lesion density can be challenging for some lesions. Thus, the flowcharts have been designed to have overlap such that in indeterminate cases, either chart will result in the same diagnosis/recommendation. For MRI, we incorporate chemical shift imaging to identify microscopic fat within the lesion (Fig. 5) and post-contrast imaging to distinguish cyst-like lesions from solid lesions. In each algorithm, we have also incorporated whether the patient has a history of known malignancy with propensity to bone metastases (i.e., kidney, prostate, breast, lung, thyroid, assuming that the patient has had appropriate screening for these malignancies, such as prostate-specific antigen or screening mammography) and/or the presence of pain attributable to the lesion, as these clinical features would make the lesion more suspicious for malignancy.

Many of these incidental lesions seen on MRI or CT will be performed to evaluate painful conditions such as trauma, muscle injuries, stress fractures, or internal joint derangement and are often unrelated to the working clinical diagnosis, and orthopedic consultation may be required to clarify the source of pain. For example, if an incidental bone lesion is found on a shoulder MRI in a patient with a rotator cuff tear, the patient’s pain may be due to the lesion but could also be due only to the rotator cuff tear. Many entities can have variable imaging appearance and so could be included in more than one or even all three charts. Lastly, we want to stress that radiographs can be helpful in lesion assessment especially for incidental lesions discovered on MRI and should be taken into account if available.

### Solitary lucent lesion on CT

**Fig. 6:**
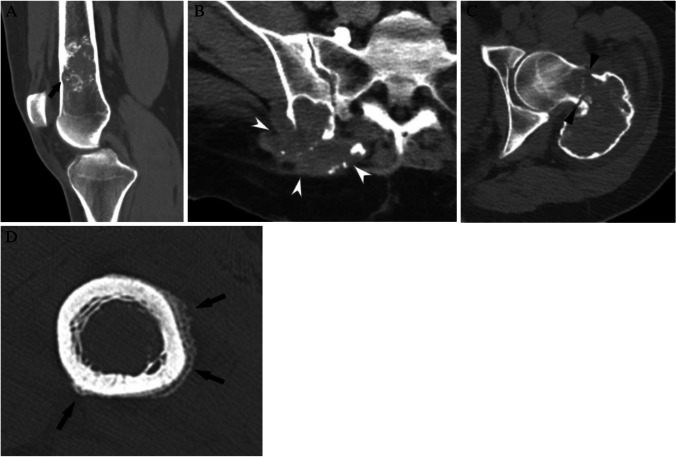
CT assessment of concerning features. **A** Coronal CT image shows cortical involvement (arrow) of the anterior femoral cortex from an enchondroma. **B** Axial soft tissue window CT image shows a destructive lung cancer metastasis in the ilium with large soft tissue component (pointed arrows). **C** Axial CT image shows a pathologic fracture (arrowheads) in the femoral neck due to a giant cell tumor of bone. **D** Axial CT image shows periosteal reaction (thin arrows) along the femoral cortex from presumed stress fracture

The initial evaluation of a solitary lucent lesion on CT should begin with an assessment for concerning clinical (pain attributable to the lesion) or imaging (cortical involvement, soft tissue extension, pathologic fracture, or aggressive periosteal reaction) features [25]. The presence of any of these concerning features (Fig. 6) results in a Bone-RADS4 designation, prompting biopsy and/or oncologic referral for further workup. The term cortical involvement would include lesions with cortical tunneling, endosteal scalloping, bone remodeling/expansion, and cortical thickening. Lesions with these concerning features could include bone metastases, multiple myeloma, Langerhans cell histiocytosis, and osteomyelitis. While some features, particularly cortical involvement, may seem equivocal, referral for further evaluation with an orthopedic oncologist is often a safe and reasonable choice, as benign lesions, such as simple bone cysts, may also require treatment or prophylactic fixation to prevent pathological fracture.

For lesions without concerning features, the next step is to determine if the patient has a known malignancy with propensity to metastasize to the bone. If present, further evaluation with FDG-PET, bone scan, or MRI for further characterization (Bone-RADS2) or follow-up imaging (Bone-RADS3) to evaluate for interval change is recommended to minimize the risk of missing a new metastasis. It is important to restate that the algorithms only apply for solitary lesions and not multiple lesions.

If there are no concerning features or patient history of malignancy, the next step is to evaluate for significant fat in the lesion as several benign entities contain fat. Confirmation of the presence of fat can be directly visualized as macroscopic fat *or* determined by using a region of interest (ROI) placed in the lesion that measures with fat attenuation (mean density less than − 10 HU). If internal fat in the lesion is definitively confirmed, then the lesion is designated a Bone-RADS1, and no additional workup is needed. Intraosseous lipoma, hemangioma, and red marrow would be representative lesions.

**Fig. 7:**
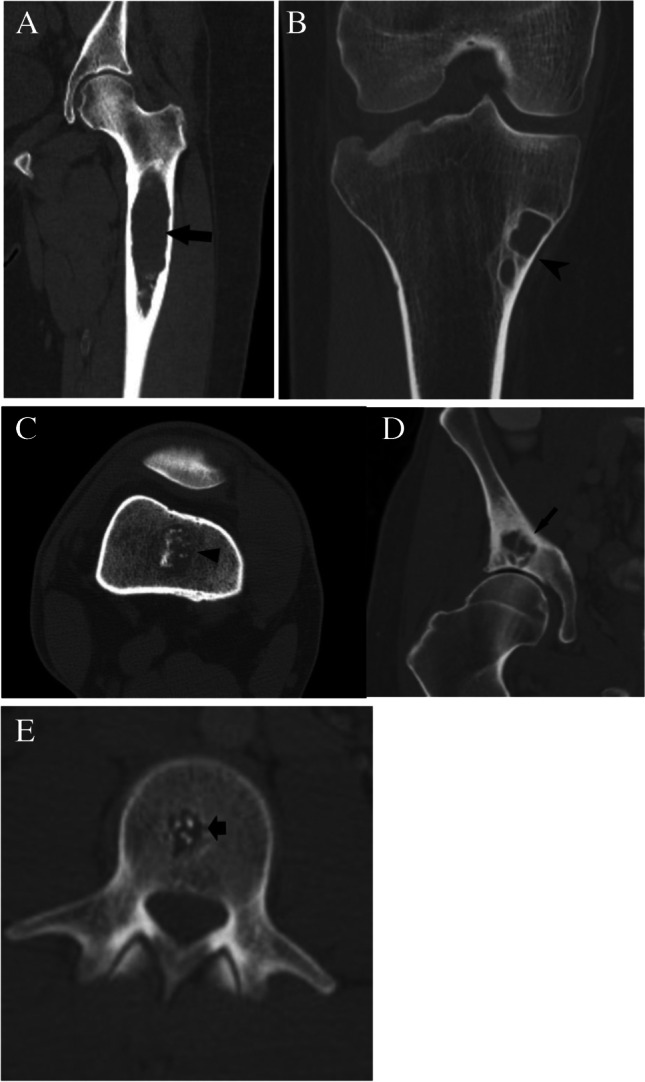
Characteristic CT lucent lesions. **A** Coronal CT image shows a lucent lesion (arrow) in the proximal femur consistent with fibrous dysplasia, **B** coronal CT image shows a cortically based lucent non-ossifying fibroma in the proximal tibia, **C** coronal CT image shows an enchondroma with punctate calcifications in the distal femur, **D** coronal CT image shows a subchondral cyst abutting the right hip joint with degenerative changes, and **E** axial CT image shows a lucent lesion with a “polka dot” appearance of calcifications in T12 consistent with a hemangioma

Next, it is helpful to identify any benign lesions that can be recognized on CT with high confidence, specifically those with well-established characteristic features, such as fibrous dysplasia, non-ossifying fibroma, enchondroma, subchondral cyst, and hemangioma (Fig. 7). If a lesion is recognized as entirely consistent with any of these five benign entities, it is a Bone-RADS1 and requires no further workup. It should be reemphasized that if the lesion has concerning features such as endosteal scalloping in an enchondroma located in a long bone, then that lesion would have been designated a Bone-RADS4 at the beginning of the algorithm.

Any lesion that is not recognized as belonging in the Bone-RADS1 management category would require further workup. The urgency of the workup is based on the imaging features. For cortically based lesions, radiologists should assess for features suggesting an osteoid osteoma, osteoblastoma, or cortical metastasis. Medullary lucent appearing lesions encompass a variety of benign and malignant entities, including aneurysmal bone cyst, unicameral bone cyst, giant cell tumor, chondroblastoma, clear cell chondrosarcoma, myeloma, or metastasis. All of these lesions should be classified as Bone-RADS4 and referred to oncology and/or biopsy to prompt definitive management. If the lesion lacks clear features, it should either undergo additional workup with FDG-PET, bone scan, or MRI or be followed with additional imaging in 6 months.

### Solitary sclerotic/mixed density lesion on CT

Similar to the approach of a lucent lesion, the initial evaluation of a sclerotic/mixed density lesion on CT should begin with an assessment for concerning clinical (pain attributable to the lesion) or imaging (cortical involvement, soft tissue extension, pathologic fracture, or aggressive periosteal reaction) features. The presence of any of these concerning features results in a Bone-RADS4 designation and would include metastases, myeloma, chondrosarcoma, or infection. If the lesion lacks concerning features, then the next step is to determine if the patient has a known malignancy with propensity to metastasize to the bone. If there is a malignancy history, then further evaluation of the solitary lesion with FDG-PET, bone scan, or MRI for further characterization (Bone-RADS2) or follow-up imaging (Bone-RADS3) is warranted as with the lucent lesion CT algorithm.

For lesions without concerning features and in a patient without a known malignancy with propensity to metastasize to the bone, the lesion should be assessed for whether it has any features that are highly characteristic of a benign sclerotic bone lesion, such as non-ossifying fibroma, enostosis, osteoma, or sclerosing bone dysplasia. If the classic features for one of these benign entities are present, the lesion can be designated Bone-RADS1 and left alone. Regarding enostosis, studies have shown that if a purely sclerotic lesion demonstrates a mean HU > 885, this is sensitive and specific for enostosis [18, 20, 25, 26]. However, in the setting of an older patient, reliance on thresholds should be limited. Interestingly, a recent study from Hong et al. showed that expert readers and radiomics models outperform strict HU thresholds[27].

If the lesion is not purely sclerotic, the presence of internal fat should be assessed. If present, then the lesion is designated a Bone-RADS1 and can be left alone. Differential considerations when internal fat is present include intraosseous lipoma, hemangioma, Paget disease, osteonecrosis, fibrous dysplasia, or non-ossifying fibroma (NOF).

If no fat is present, the lesion should then be assessed for “ground glass” appearance of fibrous dysplasia. The term “ground glass” has become synonymous with fibrous dysplasia in skeletal lesions, but it probably found its origins in thoracic radiology, where it described a diffusely hazy and smooth appearance. The range of densities varies widely, from sclerotic to lucent [28]. If present, the lesion is designated Bone-RADS1, and no additional management is needed. Most fibrous dysplasia is isolated, but if there are endocrine abnormalities or other sites of skeletal deformity, consider bone scan or skeletal survey to look for other sites of disease.

If it does not have a “ground glass” appearance, the lesion should next be assessed for cartilage matrix which appears as punctate calcifications often as small arcs and rings. If cartilage matrix is present, one should determine whether the lesion has any imaging features suggesting aggressive growth, which include endosteal scalloping, expansile remodeling, cortical breakthrough, periosteal reaction, and/or soft tissue mass. For endosteal scalloping in a long bone, involvement of greater than two-thirds of the cortical thickness or extension of scalloping greater than two-thirds of the craniocaudal length of the lesion raise suspicion for malignancy [29]. An additional feature that may raise concern is incomplete mineralization because malignant cartilaginous lesions more often show areas that are not mineralized as compared to non-aggressive cartilaginous lesions (case 4, Fig. 14) [30, 31]. However, this should be considered in the context with the other features of the lesion and not as a stand-alone feature of aggression. Similarly, studies have shown overlap in the size range of enchondromas and chondrosarcomas of long bones [29, 32]. Therefore, size should not be used as a stand-alone discriminator for aggressiveness. Although rare, an axial skeleton location, such as the pelvis, ribs, and sternum, is also a concerning feature because enchondromas are very rare (< 1%) in the flat bones [33]. In fact, in the authors’ experience, axial lesions are generally referred to as low-grade chondrosarcomas during radiology-pathology correlation even when they lack aggressive imaging features. However, because low-grade lesions without aggressive growth features carry a good prognosis, these can be treated with close clinical and imaging follow-up or with intralesional curettage [34]. Lastly, an epiphyseal location raises concern for malignancy due to the predilection of clear cell chondrosarcoma in this location. If any of these concerning features are present, the cartilage lesion is designated a Bone-RADS4, and representative lesions include atypical cartilaginous tumor/grade I chondrosarcoma, chondrosarcoma, and chondroblastoma. If there are no concerning features, the cartilage forming lesion is presumed to be an enchondroma and can be deemed Bone-RADS1 and left alone. Due to the low rate of chondrosarcoma in incidental painless cartilaginous lesions of the long bones, routine follow-up is considered unnecessary and can be instead reserved for when new symptoms arise[32, 35, 36].

### Assessment of lesion density

**Fig. 8:**
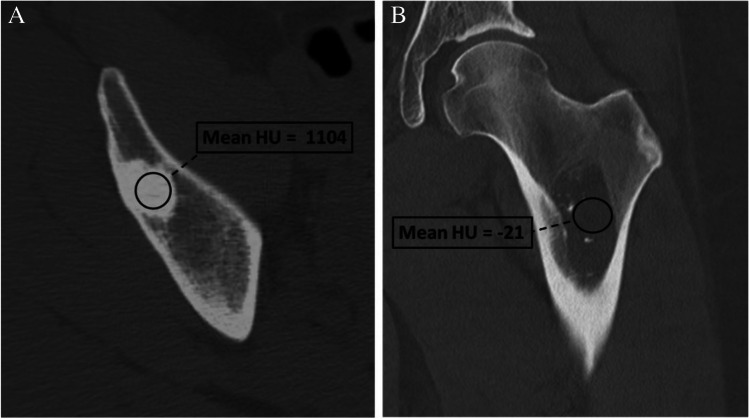
Assessment of lesion density with ROI. We recommend placing a ROI in the largest area that is most representative of the lesion to obtain mean Hounsfield unit (HU) values. **A** Incidental sclerotic focus in the right iliac bone has mean HU = 1104 consistent with an enostosis. **B** Lesion in the proximal femur has mean HU =  − 21 consistent with fat in an intraosseous lipoma

One of the advantages of CT in evaluating bone lesions is its ability to measure relative density. Lesions that contain fat or are extremely dense are often benign [18, 20]. However, there is more than one way to assess lesion density (e.g., mean density, maximum density), and these values may vary depending on where the region of interest (ROI) is placed within the lesion (Fig. 8). Bone density measurements can vary slightly from scanner to scanner, with an accepted precision of approximately 3–5%, which is improved with the use of dual energy scanners over single photon scanners [37]. Intravenous iodinated contrast can elevate lesion density and even simulate a bone lesion [38], so values should be assessed on non-contrast images when possible.

### Solitary lesions on MRI

#### High T1 lesion

MRI can detect incidental bone lesions which are occult or poorly seen on radiographs or CT. When evaluating a bone lesion on MRI, one should begin with the T1 signal characteristics. If a T1 sequence is not available, the patient should return for dedicated T1-weighted imaging

##### T1 signal much higher than skeletal muscle or adjacent intervertebral disc

Lesions with internal high T1 signal that matches subcutaneous fat signal on all pulse sequences are almost always benign (Bone-RADS1) [20]. The differential considerations are focal fatty marrow, intraosseous hemangioma, lipoma, osteonecrosis, Paget disease, Modic type 2 discogenic degenerative endplate changes, and other post-inflammatory focal marrow atrophy [39, 40]. When high T1 signal in a lesion is not the same as subcutaneous fat signal on all pulse sequences, both benign and malignant lesions are considerations. Here, post-contrast imaging can then help further narrow the differential diagnosis. If a T1 hyperintense lesion shows no or only thin peripheral enhancement, an intraosseous ganglion or subchondral cyst containing proteinaceous material should be considered, particularly when in a periarticular location (Bone-RADS1). Conversely, a T1 hyperintense lesion with central or mass-like enhancement would warrant a Bone-RADS4 designation, with differential considerations including melanoma metastasis or hemorrhagic metastasis. Of note, internal enhancement within intraosseous ganglia or subchondral cysts can be related to internal fibrous component, enhancing synovial joint fluid entering the cyst, or contrast diffusion into the cyst from surrounding bone marrow [41]. Melanoma metastases can present as well-circumscribed high T1 lesions owing to the paramagnetic effect of melanin and presence of hemorrhage and show solid enhancement on post-contrast images [42]. If post-contrast images are not available, a high T1 lesion not matching the subcutaneous fat signal is considered a Bone-RADS2 lesion, and additional imaging with contrast or chemical shift sequences is recommended for further characterization. On chemical shift images, a signal drop of > 20% supports a benign diagnosis, while a signal drop of less than or equal to 20% is indeterminate and cannot differentiate between benign and malignant neoplasms. Using 20% signal drop as a cutoff has a 95–100% sensitivity and 61–95% specificity[43–47].

##### T1 signal slightly higher than skeletal muscle or adjacent intervertebral disc

Focal islands of red marrow are the classic lesion in this category and are common diagnostic dilemmas. One should search for the presence of macroscopic internal fat in the lesion which is highly predictive of benignity [20]. Red marrow can have concerning MRI appearance as some lesions can be mass-like and may not show evidence of macroscopic fat. For these equivocal areas of red marrow, chemical shift imaging with in-phase and out-of-phase T1-weighted gradient images may be helpful in differentiating between red marrow from neoplastic processes [48]. If chemical shift imaging is not available, these slightly high T1 lesions are considered Bone-RADS2, and additional chemical shift imaging, radiographs, and/or bone scan should be performed. The red marrow lesion would be normal on plain film radiograph, and the bone scan would not show uptake. A focal marrow abnormality that fails to show macroscopic or microscopic fat mandates further investigation, and we recommend applying the “low T1” algorithm to these lesions.

##### High T1 signal in fluid level or due to hemorrhage

**Fig. 9:**
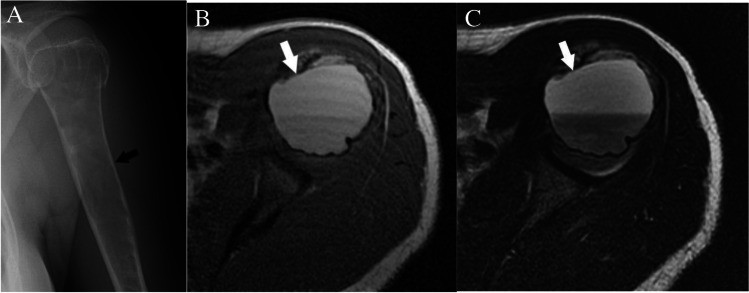
T1 hyperintense hemorrhagic lesions can mask underlying lesions and should follow the “High T2” algorithm. There is a lucent lesion (arrows) in the proximal humerus on the radiographs (**A**). The lesion has high signal (from hemorrhage) and a fluid level on the axial T1- (**B**) and axial T2- (**C**) weighted images. Lesion was was solitary bone cyst at definitive treatment

Hemorrhagic lesions with T1 bright areas can mask the underlying lesions and prevent optimal assessment and deserve special mention (Fig. 9). We recommend following the “High T2” algorithm for lesions with intralesional high T1 signal attributed to fluid–fluid or fluid-blood level, as these lesions most likely have simultaneous high T2 signal, and the hemorrhage could be masking an underlying lesion.

#### Low T1 lesion

If lesion shows low T1 signal, the next step is to evaluate T2 signal characteristics.

#### Low T1 high T2 lesions

Many neoplastic diseases are characterized by low to intermediate signal intensity on T1 and high signal intensity on T2 when compared to normal muscle [39, 49]. When detected, both primary benign and malignant bone tumors as well as metastatic lesions should be considered [50, 51]. Although MRI cannot accurately differentiate between the benign and malignant lesions solely based on signal characteristics, the incorporation of other imaging features can help in the diagnostic management of these lesions.

##### Presence of concerning imaging features

**Fig. 10:**
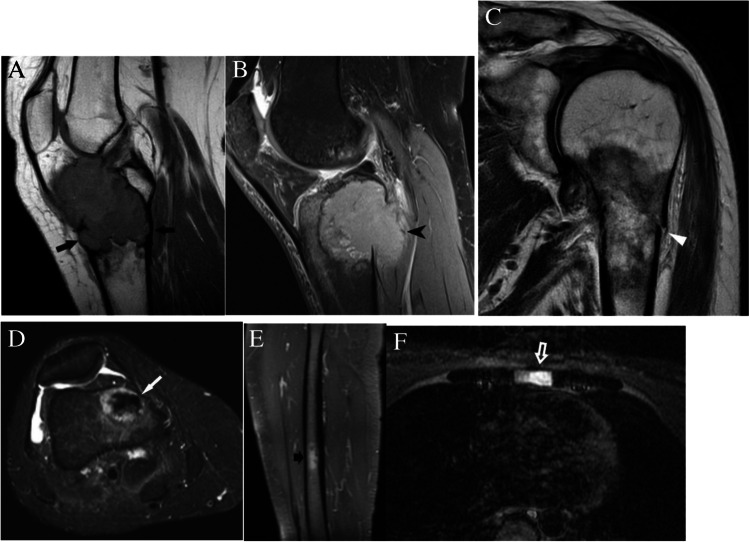
MRI assessment of concerning features. **A** Sagittal T1-weighted MR image shows a giant cell tumor in the proximal tibia with anterior and posterior cortical involvement (arrows). **B** Sagittal T2-weighted fat-suppressed MRI image shows a renal cell metastasis in the proximal tibia with soft tissue extension (pointed arrow) posteriorly. **C** Coronal T2-weighted MR image shows a lung cancer metastasis in the proximal humerus with pathologic fracture (arrowhead) of the lateral cortex. **D** Axial T2-weighted fat-suppressed MR image shows a low signal prostate cancer metastasis in the distal femur with peripheral halo (thin arrow) of high T2 signal. **E** Sagittal T1-weighted fat-suppressed post-contrast MRI image shows an enhancing breast cancer metastasis (fat arrow) in the mid shaft of the femur. **F** Axial T2-weighted fat-suppressed MR image shows a hyperintense metastasis (outlined arrow) in the sternum in a patient with breast cancer

When faced with a low T1 and high T2 incidental bone lesion, a search for concerning imaging features should be a primary focus. Presence of any of the following concerning clinical or imaging findings would result in a Bone-RADS4 designation: history of malignancy with propensity for bone metastases, pain attributable to the lesion, cortical involvement, soft tissue extension, pathologic fracture, surrounding bone marrow edema, solid mass-like enhancement, a lesion in the sternum in a patient with breast cancer (Fig. 10), or elevated prostate-specific antigen (PSA) [52].

##### Absence of concerning imaging features

If a low T1 and high T2 lesion does not have any of the above-listed concerning features, it should be assessed for characteristic imaging features of common benign lesions (Bone-RADS1). Enchondromas, subchondral cysts, osteochondromas, NOF, and fibrous dysplasia are representative lesions. Finally, for those low T1 and high T2 lesions without concerning features and but are not consistent with the listed common bone lesions, additional imaging evaluation (Bone-RADS2) with CT to look for internal matrix, or follow-up imaging (Bone-RADS3) with repeat MRI to confirm stability, should be considered.

### Low T1 and low T2 lesions

One of the main considerations for a low T1 and low T2 lesion is an enostosis; however, breast and prostate carcinoma can have purely osteoblastic metastases with corresponding low T1 and T2 signal on MRI. To distinguish between an enostosis and an osteoblastic metastasis, these lesions should be assessed for the concerning features: halo sign, solid mass-like enhancement, sternal lesion in a patient with breast cancer, or elevated PSA. The “halo sign” (Fig. 10d) is defined as a rim of abnormal increased T2 signal in the marrow surrounding the lesion of interest and has a reported sensitivity of 75%, specificity of 99%, and accuracy of 88% of being a metastasis [20]. In other words, for lesions that have the “halo sign,” 75% are metastases. Conversely, the “halo sign” is very rare in benign lesions, and 99% of benign lesions do not have the “halo sign.” Therefore, its presence should prompt further workup and referral (Bone-RADS4) [20]. Intralesional solid enhancement following administration of contrast is also a concerning feature that can be seen in osteoblastic metastases [53, 54]. A solitary sternal lesion in a patient with known breast cancer has a 76% likelihood of being metastatic [55]. For this reason, these lesions should receive a Bone-RADS4 designation.

A low T1 and T2 lesion with no concerning features in patients with history of malignancy with propensity for bone metastases may require further imaging or imaging follow-up. Options include MRI with contrast to evaluate for possible enhancement, CT to measure the HU of the lesion (refer to CT sclerotic bone lesion), or bone scan. Further workup may depend on the nature of underlying malignancy; for example, probability of bone metastasis in patient with history of prostate cancer can be predicted based on the clinical staging, prostate-specific antigen (PSA) level, and Gleason score [56]. If there are no concerning imaging features and history of malignancy, a low T1 and T2 lesion is most likely a benign lesion (Bone-RADS1), such as an enostosis, or an involuted NOF.

## Sample lesions

In this section, we present sample cases to illustrate the usability of the CT and MRI algorithms in arriving at an actionable management recommendation. Many of these lesions have characteristic imaging features that can make their diagnosis pathognomonic or have a high probability of having a benign etiology. Other lesions described here deserve additional discussion as their management can be controversial.

### *Case 1 (Fig. **11**)*

**Fig. 11:**
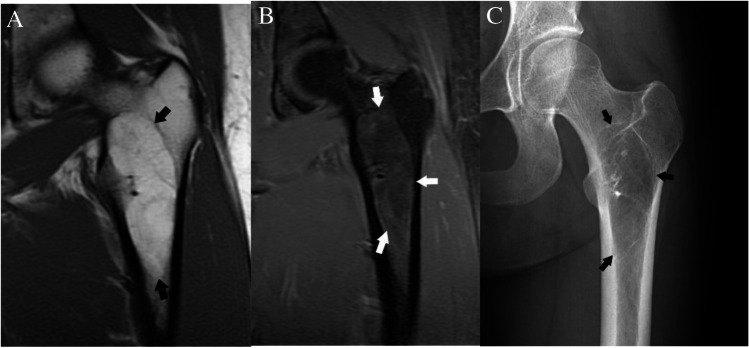
Case 1. Intraosseous lipoma. 23-year-old female runner presents with 3 months of left hip pain radiating to the groin with incidental lesion (arrows) detected in the proximal femur on MRI. The lesion is hyperintense (similar to subcutaneous fat) on the A coronal T1-weighted MR image and is hypointense on the B coronal T2-weighted fat-suppressed MR image. C Radiograph shows a lucent lesion in the proximal femur

#### *Clinical history*

A 23-year-old female runner presents with 3 months of left hip pain radiating to the groin. The patient has no major medical issues and no history of malignancy. MRI of the left hip revealed a small labral tear (not shown) and a 7 cm lesion in the proximal femur.

#### *Lesion assessment*

Using the MRI algorithm, this lesion would be classified as a high T1 lesion. Further assessment of the high T1 signal shows that the lesion follows subcutaneous fat on all MRI sequences. This leads to a designation of Bone-RADS1. The differential diagnosis includes focal fat, intraosseous lipoma, hemangioma, Paget disease, and osteonecrosis, which are all benign processes and do not warrant additional workup unless there is pain or risk for fracture. There are small foci of low signal within the lesion indicative of dystrophic calcification, and the lesion represents an intraosseous lipoma.

#### *Diagnosis and discussion*

Intraosseous lipoma is the most common lipogenic tumor in the bone and contains macroscopic fat on CT (mean density less than − 10 HU) and MRI [24]. It is most common in the proximal femur, where there is a paucity of trabecular bone. They are typically intramedullary but may be intra-cortical on rare occasions [24]. Their reported incidence (0.1%) is likely higher, as they can be confused with other lesions such as fibrous dysplasia and may be difficult to distinguish from normal fatty marrow [24, 57]. Furthermore, central areas of fluid attenuation may be due to cystic degeneration or remnants of an involuting simple bone cyst [58, 59]. It is important to highlight that identifying fat within a bone lesion has a very high negative predictive value for malignancy of 99.5% [20]. Thus, even if one is unable to clearly determine that the lesion is an intraosseous lipoma, the presence of internal fat can be highly suggestive of a benign process.

### *Case 2 (Fig. **12**)*

**Fig. 12:**
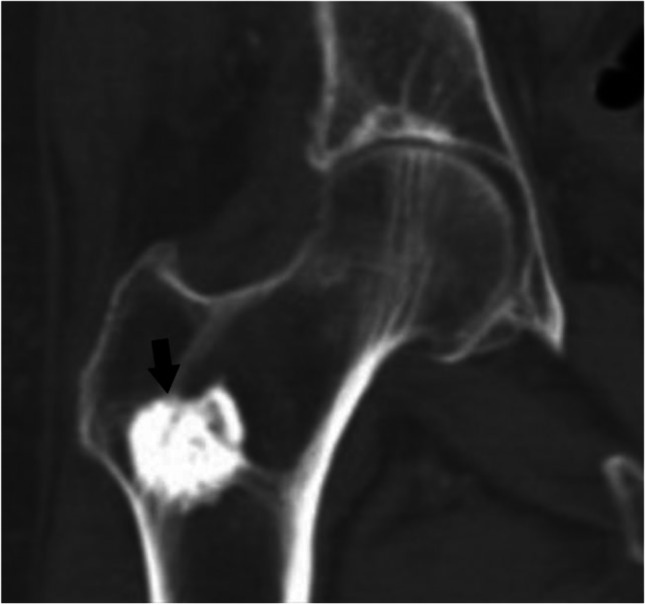
Case 2. Enostosis. 68-year-old female presents with right pelvic pain after a fall. Coronal CT image shows a densely sclerotic lesion (arrow) with spiculated margins in the proximal femur with mean density = 1644 HU

#### *Clinical history*

A 68-year-old female presents with pelvic pain after a fall. The patient has no history of malignancy. CT of the pelvis revealed left sacral fracture (not shown) and an incidental lesion in the proximal right femur (mean CT density = 1644 HU).

#### *Lesion assessment*

Using the CT algorithm for sclerotic/mixed lesions, this solitary lesion lacks aggressive features and does not have a ground glass or cartilage matrix. The mean attenuation is well above 885 HU, and it has spiculated margins typical of an enostosis. Therefore, Bone-RADS1 can be safely assigned.

#### *Diagnosis and discussion*

An enostosis is considered an asymptomatic hamartomatous focus of cortical bone in the cancellous bone which appears as a homogenous dense oval lesion with thorny or spiculated margins on radiographs and CT. On MRI, an enostosis demonstrates low T1 and T2 signal intensity similar to cortical bone on all pulse sequences. Of note, up to 31% of enostoses may grow on serial imaging even after skeletal maturity, and the growth in size is expected to be less than 25% over 6 months or 50% in 1 year [36, 59]. CT attenuation measurements can be used to distinguish enostoses from untreated osteoblastic metastases. A mean attenuation of 885 HU and a maximum attenuation of 1060 HU have been identified as reliable thresholds above which an enostosis is the favored diagnosis [18]. Of note, these HU thresholds are meant to apply only for differentiation of enostoses from untreated osteoblastic metastases and should not be generalized to all sclerotic or mixed density lesions [60].

### *Case 3 (Fig. **13**)*

**Fig. 13:**
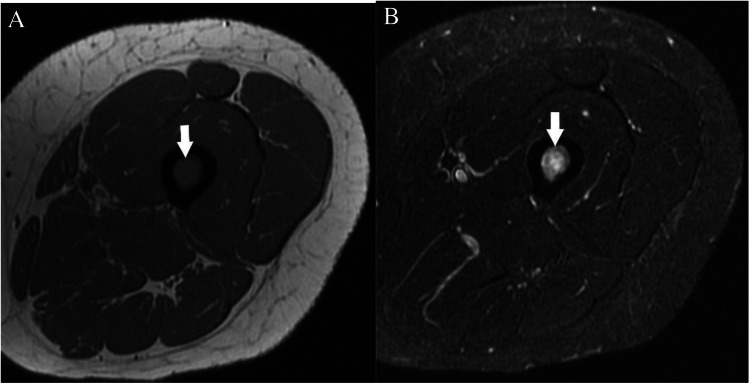
Case 3. Breast cancer metastasis. 57-year-old female runner presents with thigh pain. There is a lesion (arrows) in the midshaft of the femur that is isointense to skeletal muscle on the A axial T1-weighted MR image and hyperintense on the B axial T2-weighted fat-suppressed MR image

#### *Clinical history*

A 57-year-old female runner presents with thigh pain. MRI was performed to assess for stress fracture. Patient also had a recent diagnosis of breast cancer. Subsequent biopsy revealed metastatic breast cancer.

#### *Lesion assessment*

Images demonstrate an intramedullary lesion in the femoral shaft which is isointense to the skeletal muscle on T1-weighted and high signal on the T2-weigthed images. Following the MRI algorithm for evaluation of solitary bone lesions, solid enhancement (not shown) and history of malignancy place this lesion in the Bone-RADS4 category.

#### *Diagnosis and discussion*

Metastasis is the most common osseous lesion in the elderly. Preferred sites of metastatic deposition are the vertebrae, pelvis, ribs, and the proximal ends of long bones, owing to their high red marrow content. Metastases demonstrate a wide range of imaging manifestations from lytic to mixed lytic-sclerotic to purely sclerotic, depending on the nature of the primary neoplasm. Prostate cancer, breast cancer, transitional cell carcinoma, carcinoid, mucinous adenocarcinoma of the gastrointestinal tract, lymphoma, medulloblastoma, and neuroblastoma osseous metastases often demonstrate an osteoblastic nature and classically appear as round well circumscribed nodular lesions [16, 61]. Conversely, osteolytic lesions typically demonstrate trabecular thinning and ill-defined margins. In both cases, multiplicity provides greater specificity to the diagnosis of osseous metastatic disease. In comparison to the primary bone tumors, metastases tend to incite less periosteal reaction. Lytic metastases usually manifest as foci of low T1 and high T2 signal intensity with increased enhancement, whereas osteoblastic lesions demonstrate low signal on both T1- and T2-weighted images. A halo of increased T2 signal surrounding the low signal central lesion is suggestive of metastatic disease [20].

### *Case 4 (Fig. **14**)*

**Fig. 14:**
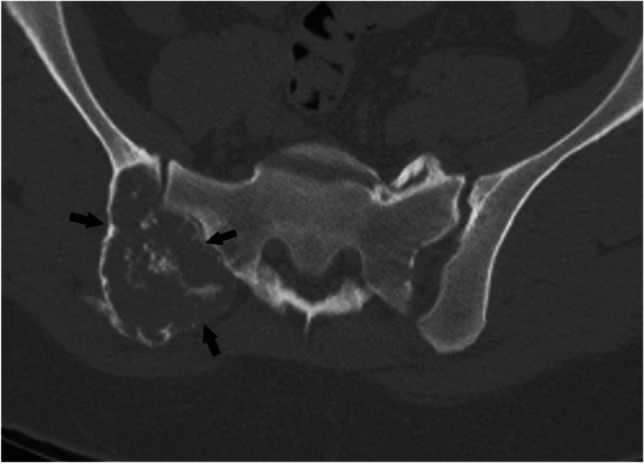
Case 4. Cartilaginous neoplasm. There is an expansile mixed lesion (arrows) expanding the posterior right iliac bone (axial location). The lesion has central punctate foci of calcifications consistent with a cartilaginous lesion and concerning endosteal scalloping. Definitive surgery revealed low-grade chondrosarcoma

#### *Clinical history*

A 54-year-old woman presents with right back pain with no history of malignancy.

#### *Lesion assessment*

There is an expansile mixed lesion replacing the posterior right iliac bone. The lesion has central punctate foci of calcifications consistent with a cartilaginous lesion. Following the CT algorithm for mixed and sclerotic lesions, this is a cartilage lesion with concerning features, axial (central) location, and endosteal scalloping. This warrants a Bone-RADS4 designation with referral to orthopedic oncology or biopsy. For this patient, the biopsy revealed a low-grade chondrosarcoma, and definitive surgery was performed.

#### *Diagnosis and discussion*

Cartilaginous neoplasms are often first discovered in the third and fourth decades of life. Common locations include the short tubular bones, femora, and humeri. They present as well-circumscribed lytic meta-diaphyseal lesions with “ring and arc” internal chondroid matrix and mild cortical scalloping. Lesions of the hand and foot often lack matrix mineralization [62]. The high water content of the cartilaginous matrix accounts for the high signal of the enchondromas on fluid-sensitive MRI sequences, whereas matrix mineralization appears as scattered low signal foci. Differentiating enchondromas from chondrosarcoma may be challenging. Chondrosarcomas are rare in the hands and feet, whereas they are common in the axial skeleton. The presence of pain, endosteal scalloping greater than two-thirds of cortical thickness, cortical destruction, periosteal reaction, soft-tissue mass, axial location, and high uptake on bone scintigraphy favor a diagnosis of chondrosarcoma [29].

### *Case 5 (Fig. **15**)*

**Fig. 15:**
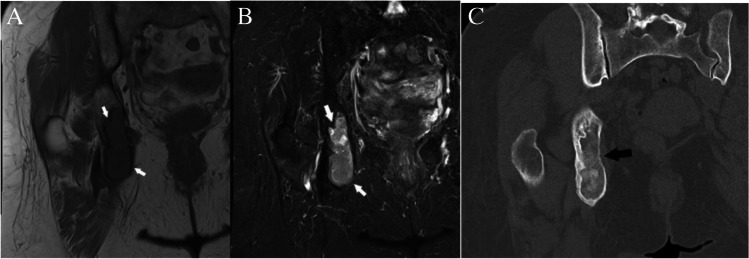
Case 5. Fibrous dysplasia. 84-year-old female with right hip pain and clinical suspicion for hip osteoarthritis who underwent right hip MRI and subsequent CT for incidentally discovered lesion (arrows) in the right ischium. The lesion is isointense to muscle on the A coronal T1-weighted MR image and hyperintense on the B coronal T2-weighted fat-suppressed MR image. The lesion has a sclerotic rim and ground glass appearance on the C coronal CT image

#### *Clinical history*

An 84-year-old female presents with right hip pain and clinical suspicion for hip osteoarthritis who underwent right hip MRI and subsequent CT. The patient had no history of malignancy.

#### *Lesion assessment*

Following the diagnostic algorithm of incidental lesions on MRI, this low T1 and high T2 signal lesion with no worrisome imaging feature in a patient without known malignancy falls into Bone-RADS 1–3 categories, depending on the reader’s experience. In this case, a Bone-RADS2 designation was applied, and the patient underwent subsequent CT. The characteristic ground-glass matrix seen on CT allowed for a final diagnosis of fibrous dysplasia (Bone-RADS1).

#### *Diagnosis and discussion*

Fibrous dysplasia is a non-inherited monostotic (70%) or polyostotic anomaly of bone formation where the normal bone is replaced with immature fibro-osseous connective tissue. It can be identified at any age, most commonly between 2 and 30 years. The most common sites are the ribs, proximal femurs, and craniofacial bones. The classic radiographic and CT appearance is a well-defined lucent expansile lesion with a sclerotic rim and the characteristic “ground glass” internal matrix. Endosteal scalloping, bowing deformities, and secondary aneurysmal bone cysts may be seen [62, 63]. On MRI, FD is of low signal intensity on T1- and a variable (intermediate to high) appearance on T2-weighted images.

### *Case 6 (Fig. **16**)*

**Fig. 16:**
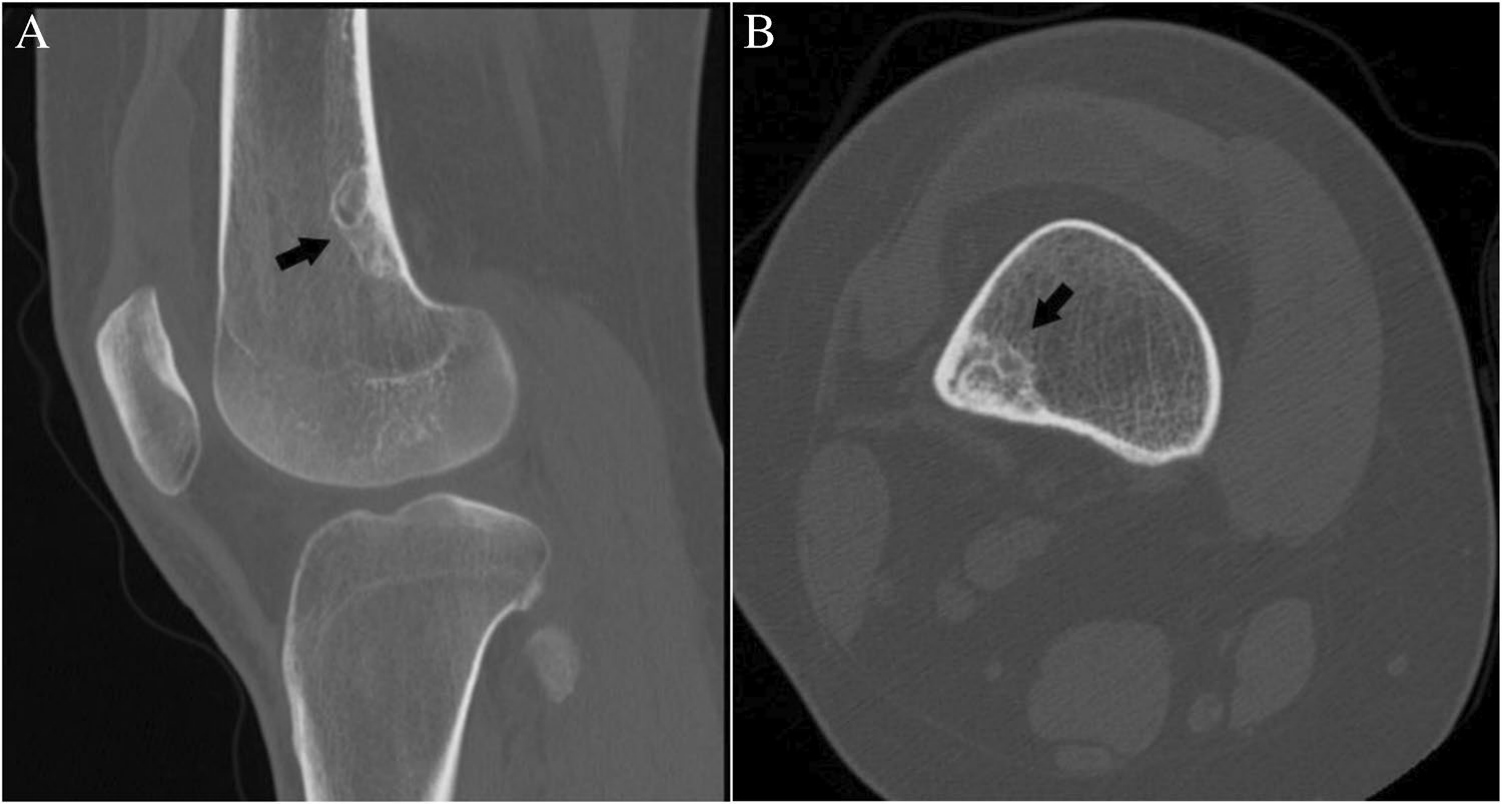
Case 6. Non-ossifying fibroma. 21-year-old man with medial knee pain. A Sagittal and B axial CT images show a mixed density cortically based lesion (arrows) with in the distal femur without concerning features

#### *Clinical history*

A 21-year-old man presents with medial knee pain. CT scan reveals a joint effusion and an incidental lesion in the lateral femoral metaphysis.

#### *Lesion assessment*

Following the CT algorithm for mixed or sclerotic lesions, this solitary lesion has no aggressive features that lack chondroid or ground glass matrix. After determining that its attenuation is less than 885 HU, the algorithm leads the user to ask whether the lesion could be consistent with either NOF, enostosis, and sclerosing bone dysplasia. In this case, the lesion is most consistent with NOF.

#### *Diagnosis and discussion*

Non-ossifying fibromas (NOF) are benign lesions of fibrous origin which are often found incidentally in the metaphysis of the long bones of the pediatric patients. Characteristic CT appearance is a cortically based lucent lesion with lobulated edges and surrounding sclerotic bone. Healing occurs as the lucent portion fills in with normal bone during adolescence, after which they can appear as an area of benign sclerosis with mild expansion. MRI appearance is variable depending on the developmental stage of the lesion. Early in its development, the lesion is of high signal on T2-weighted images. With maturation and formation of fibrous tissue, foci of low signal intensity appear, resulting in a combination of high and low signal intensity components [62, 64].

### *Case 7 (Fig. **17**)*

**Fig. 17:**
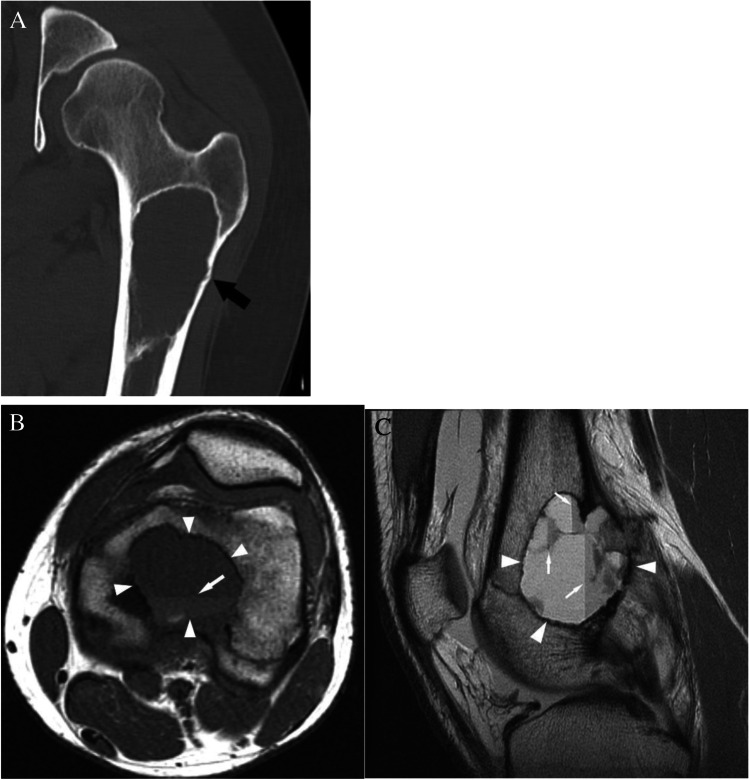
Case 7. A 27-year-old female presents with vague thigh pain for 1 year. Coronal CT image reveals a solitary focal lucent lesion in the left proximal femoral metaphysis with a well-defined sclerotic margin, cortical involvement (arrow), and mean HU of 18 units; a simple bone cyst at surgery. B and C 18-year-old female presents with knee pain worse over last 3 months. MRI of the left knee reveals a cystic lesion (arrowheads) involving the distal femoral metaphysis with fluid levels (thin arrows) due to internal blood products. The lesion has a well-defined sclerotic margin and demonstrates mild expansile remodeling. The lesion was aneurysmal bone cyst at treatment

#### Case 7A

##### *Clinical history*

A 27-year-old female presents with vague thigh pain for 1 year. The patient has no history of malignancy. CT reveals a solitary focal lesion in the left proximal femoral metaphysis with a well-defined sclerotic margin.

#### Case 7B

##### *Clinical history*

An 18-year-old female presents with knee pain worse over last 3 months. The patient has no major medical issues and no history of malignancy. MRI of the left knee reveals a cystic lesion involving the distal femoral metaphysis with fluid levels compatible with internal blood products. The lesion has a well-defined sclerotic margin and demonstrates mild expansile remodeling.

##### *Lesion assessment*

Cases 7A and 7B follow the solitary lucent lesion algorithm. Following the solitary lucent lesion algorithm, the lesion does not have any concerning features and is not in a patient with known malignancy. The lesion does not have a mean density <  − 10 HU, and its imaging features are not compatible with fibrous dysplasia, a non-ossifying fibroma, an enchondroma, a subchondral cyst, or a hemangioma. The imaging features are very characteristic of a solitary bone cyst, and its cortical thinning also places it at risk for pathologic fractures. Thus, the lesion should be in the Bone-RADS4 category with orthopedic consultation suggested.

Case 7B would follow the solitary bone lesion on MRI algorithm. It shows high signal on T1-weighted images with fluid levels and hemorrhage. It also demonstrates high T2 signal. The lesion demonstrates endosteal scalloping, cortical involvement, and expansile remodeling. This would place it in the Bone-RADS4 category. The imaging features are consistent with an aneurysmal bone cyst, and orthopedic consultation is suggested.

##### *Diagnosis and discussion*

These two similar cases depict differing forms of benign lytic lesions that can arise in the osseous structures. Solitary bone cysts are benign lytic lesions occurring primarily in children which are thought to result from a defect in bone growth that fills with fluid. They often occur in the long bones adjacent to the growth plate and are typically asymptomatic unless a pathologic fracture occurs [64]. Aneurysmal bone cysts occur predominantly in children and adolescents and are cystic lesions composed of blood-filled channels. This lesion presents as a primary or secondary lesion arising in a preexisting lesion [65].

These lesions can result in pathologic fracture or other complications, and so orthopedic consultation is warranted. These lesions follow similar courses through the solitary lucent lesion CT algorithm ending with a medullary-based lesion resulting in categorization as Bone-RADS4. Giant cell tumor of bone and unicameral bone cysts have similar MR algorithms with low T1-weighted signal and ending with an intermediate aggressive appearance which results in Bone-RADS4 classification. The aneurysmal bone cyst differs slightly as it is often a high T1 lesion; however, the presence of fluid levels redirects it to the low T1- and high T2-weighted lesions. Aneurysmal bone cysts then follow a similar path as giant cell tumors of bone and unicameral bone cysts.

### *Case 8 (Fig. **18**)*

**Fig. 18:**
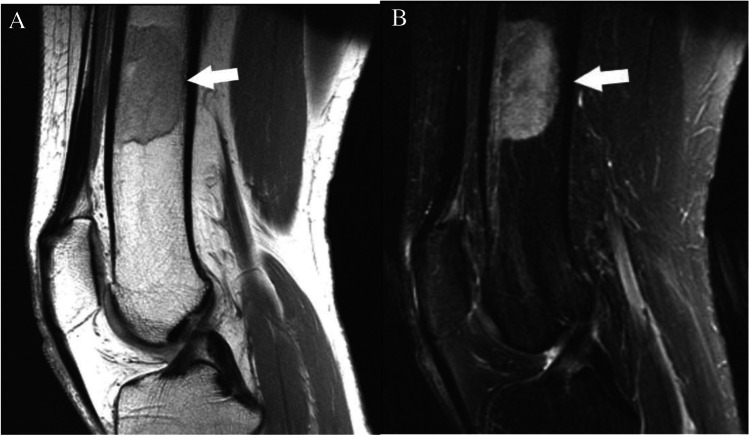
Case 8. Red marrow. 39-year-old female presents with knee pain suspicious for meniscal tear. There is an incidentally discovered lesion (arrows) in the distal femoral shaft that is slightly hyperintense to skeletal muscle on the A sagittal T1-weighted MR image and hyperintense on the B sagittal T2 fat-suppressed MR image. This finding is compatible with focal red marrow

#### *Clinical history*

A 39-year-old female presents with knee pain suspicious of meniscal tear. There is no history of malignancy or other major medical issue. MRI of the right distal femur depicts a geographic focus of signal in the distal femoral diaphysis. The lesion shows hyperintense signal on fluid-sensitive sequences. On T1-weighed images, the lesion shows hypointense signal to the surrounding fatty marrow but hyperintense signal compared to skeletal muscle. There is no endosteal scalloping or necrosis within the lesion.

#### *Lesion assessment*

Using the MRI algorithm, this lesion would fit under the high T1 lesion. The T1 signal of the femoral lesion is much higher than skeletal muscle but does not have similar signal to subcutaneous fat on all pulse sequences. No post-contrast images are available. This would place this lesion in the Bone-RADS2 category, and post-contrast or in- and out-of-phase chemical shift imaging should be considered. The imaging features suggest focal red marrow within the distal femoral diaphysis. In-and-out of phase chemical shift imaging would be very useful to document signal drop on out-of-phase images.

#### *Diagnosis and discussion*

Bone marrow is classified as red or yellow based on the fat content. Fat content in yellow marrow is approximately 80% compared to only 40% with red marrow. This accounts for the differing MR imaging appearance of red and yellow marrow. Yellow marrow, with its high fat content, shows hyperintense signal similar to subcutaneous fat, while red marrow shows T1 signal hypointense to subcutaneous fat but hyperintense signal compared to muscle and intervertebral discs [66]. Yellow marrow on fat-suppressed T2-weighted sequences shows homogenous fat suppression and is hypointense to muscle. Red marrow, containing less fat, does not homogenously suppress on fat-suppressed T2-weighted sequences and therefore shows hyperintense signal compared to muscle [66]. This hyperintense T2 signal can raise concern; however, the characteristic T1 signal typically allows recognition of red marrow. Following gadolinium administration red marrow can minimally enhance but should not avidly enhance. Yellow marrow shows no enhancement [66]. In- and out-of-phase chemical shift imaging can be useful as red marrow contains fat and decreases on out-of-phase images [48].

### *Case 9 (Fig. **19**)*

**Fig. 19:**
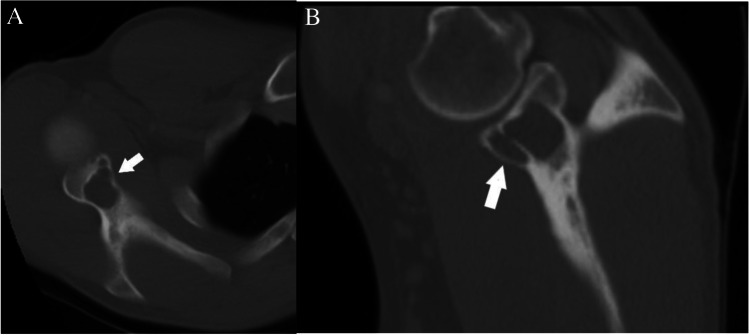
Case 9. Subchondral cyst. 30-year-old man presents with 1 month of pleuritic chest pain. There is an asymptomatic incidental lucent lesion (arrows) in the glenoid which abuts the articular surface on the A axial and B sagittal CT images. Lesion has mean density of 25 HU

#### *Clinical history*

A 30-year-old man presents with 1 month of pleuritic chest pain. The patient has no major medical issues and no history of malignancy. Chest CT reveals a lucent lesion in the right glenoid abutting the articular surface. The lesion has thin sclerotic margins with minimal endosteal scalloping.

#### *Lesion assessment*

The CT solitary lucent lesion algorithm should be utilized in this case. The lesion does not demonstrate aggressive features, and the patient does not have a history of malignancy. A ROI placed in the center of lesion measures a mean density of 25 HU, which is greater than − 10, and therefore the lesion does not contain macroscopic fat. The imaging appearance and location of this lesion abutting the articular surface of the of the glenohumeral joint would be in keeping with a subchondral cyst. Thin section CT may be helpful for locating a connection to the articular surface, if it is not readily apparent. Bone-RADS1 would be the designation in this case.

#### *Diagnosis and discussion*

Subchondral cysts present as well-defined lytic lesions abutting the articular surface. They arise in the subchondral epiphysis but may extend to involve the metaphysis. They are associated with pathology of the adjacent joint such as osteoarthritis or inflammatory arthropathy. There are two theories as to their origin [67]. The first is that the overlying articular hyaline cartilage and subchondral cortex are penetrated or breached allowing mechanical hydrolysis of the underlying subchondral bone by synovial fluid resulting in formation of an interosseus cyst. The second theory is that traumatic forces on the subchondral bone result in cellular damage. The ensuing necrosis results in cyst formation in the periarticular osseous structures. Both theories likely occur with the mechanism determined by the underlying disorder leading to the subchondral cyst. Subchondral cysts are not typically symptomatic; however, underlying joint disorders or trauma is often associated with pain, swelling, or limitation in motion.

### *Case 10 (Fig. **20**)*

**Fig. 20:**
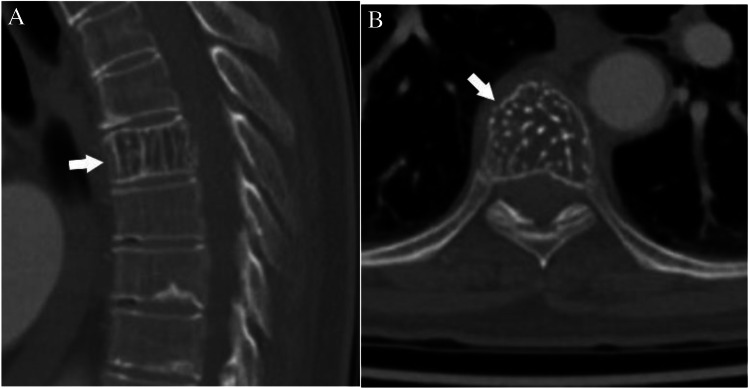
Case 10. Hemangioma. 73-year-old female with chest pain. Chest CT reveals an incidental T7 lesion. The lesion (arrows) has vertical striations on the A sagittal CT image and results in a “polka dot” appearance on the B axial CT image

#### *Clinical history*

A 73-year-old female presents with chest pain. The patient has no history of malignancy. Chest CT incidentally reveals a lucent lesion in the T7 vertebra. The lesion has areas of fatty attenuation with vertically oriented trabecula extending throughout the lesion.

#### *Lesion assessment*

The solitary mixed or sclerotic algorithm would be appropriate for this lesion. The patient has no history of malignancy. Significant portions of the lesion demonstrate macroscopic fat, which allow for a confident Bone-RADS1 designation. The differential includes intraosseous lipoma, hemangioma, Paget disease, or fibrous dysplasia. These lesions are all benign and require no further workup. Sagittal and coronal images of the lesion depict vertically oriented, thickened trabecula surrounded by fat compatible with the “corduroy sign.” Axial images demonstrated small round foci of residual bone surrounded by fat compatible with the “polka-dot sign.” The features are consistent with a hemangioma.

#### *Diagnosis and discussion*

The incidence of hemangiomas at autopsy is approximately 10% making them the most common benign neoplasm of the vertebrae [68]. They are typically found as incidental findings since the majority are asymptomatic. Hemangiomas can be found in any osseous structure but are most common in the thoracic spine [69]. They are composed of vascular spaces that displace bone creating the characteristic vertically oriented residual trabecula extending through the lesion. If incidentally found, no further workup or follow-up is required for hemangiomas.

## Controversies

At the SSR annual meeting, a recurring concern was that certain lesions with “classic” appearances to a MSK radiologist, such a fibrous dysplasia or hemangioma, can be difficult for non-MSK radiologists to definitively diagnose, resulting in a Bone-RADS4 designation based on their use of the algorithms. While we agree that excessive imaging can be harmful, we want to again emphasize that these flowcharts should never override expert opinion. They can serve as a reference point if there is uncertainty. If an MSK radiologist’s opinion in a certain situation deviates from the flowchart in a certain case, then the chart should defer to the expert opinion. It is impossible for any schematic or flowchart to address all permutations and situations. Also, we feel that dismissing a malignant lesion has greater consequences than over-imaging a benign lesion; therefore, we have erred on the side of not ignoring Bone-RADS4 lesions.

## Conclusion

The Practice Guidelines and Technical Standards Committee of the Society of Skeletal Radiology (SSR) proposes a bone reporting and data system (Bone-RADS) and diagnostic algorithms for the diagnostic management of incidental solitary bone lesions seen on CT and MRI. The algorithms represent a consensus statement incorporating the current understanding of the diagnostic management of bone lesions, and we hope their use will help to improve and standardize patient care. Lastly, we expect that these algorithms may need future modification with validation studies and advances in tumor imaging.
